# Modification of radiosensitivity by Curcumin in human pancreatic cancer cell lines

**DOI:** 10.1038/s41598-020-60765-1

**Published:** 2020-03-02

**Authors:** Katharina Schwarz, Sophie Dobiasch, Lily Nguyen, Daniela Schilling, Stephanie E. Combs

**Affiliations:** 1 Department of Radiation Oncology, Technical University of Munich (TUM), Klinikum rechts der Isar, Ismaninger Straße 22, 81675 Munich, Germany; 2 Institute of Radiation Medicine (IRM), Department of Radiation Sciences (DRS), Helmholtz Zentrum München, Ingolstädter Landstraße 1, 85764 Neuherberg, Germany; 3 German Cancer Consortium (DKTK), Partner Site Munich, Munich, Germany

**Keywords:** Cell death, Cell growth, Gastrointestinal cancer, Tumour heterogeneity, Cancer

## Abstract

Pancreatic cancer is one of the most aggressive malignancies and is characterized by a low 5-year survival rate, a broad genetic diversity and a high resistance to conventional therapies. As a result, novel therapeutic agents to improve the current situation are needed urgently. Curcumin, a polyphenolic colorant derived from Curcuma longa root, showed pleiotropic influences on cellular pathways *in vitro* and amongst others anti-cancer properties including sensitization of tumor cells to chemo- and radiation-therapy. In this study, we evaluated the impact of Curcumin on the radiosensitivity of the established human pancreatic cancer cell lines Panc-1 and MiaPaCa-2 *in vitro*. In contrast to MiaPaCa-2 cells, we found a significant radiosensitization by Curcumin in the more radioresistant Panc-1 cells, possibly caused by cell cycle arrest in the most radiation-sensitive G2/M-phase at the time of irradiation. Furthermore, a significant enhancement of radiation-induced apoptosis, DNA-double-strand breaks and G2/M-arrest after curcumin treatment was observed in both cell lines. These *in vitro* findings suggest that especially patients with more radioresistant tumors could benefit from a radiation-concomitant, phytotherapeutic therapy with Curcumin.

## Introduction

Pancreatic cancer is one of the most lethal cancers worldwide. In 2018 pancreatic cancer made 2.5% of worldwide cancers^1^, but the incidence is still increasing and calculated from actual data pancreatic ductal adenocarcinoma (PDAC) may be the second leading cause of cancer-related deaths by 2030^2^. Due to high chemo- and radiation resistance, mortality is very high, and the 5-year survival rate in Germany was only about 10% in 2013. Actual the only curative therapy is a total surgical resection but caused by late and not specific symptoms pancreatic cancer is diagnosed in non-resectable disease in almost 80% of patients^3^. In an advanced state of disease, combined regimes with neoadjuvant chemotherapy or chemoradiation followed by surgery are used. Due to improvements and technical advances in recent years, e.g. Intensity-modulated radiation therapy, stereotactic radiation or intraoperative techniques, radiation therapy (RT) might be an efficient therapeutic option with less toxicity in patients with locally advanced PDAC^4^. But based on tumor heterogeneity, structure and biology novel agents are urgently needed to improve the efficacy of radiotherapy and to overcome therapy resistance.

Curcumin, a polyphenolic colourant extracted from Curcuma longa root, has been shown to possess anti-inflammatory, anti-oxidant and anti-neoplastic activity in multiple ways *in vitro*. Anti-inflammatory effects postulate a positive impact on treatment side effects like weight loss, radiation dermatitis, pneumonitis, myelosuppression or secondary tumors after radiation therapy^5^. In a phase-II-study with PDAC patients, an oral dose of 8 g Curcumin per day did not show any side effects^6^. Actual studies investigate a concordant treatment consisting of Gemcitabine and Curcumin for patients with advanced pancreatic cancer^7^. However, caused by poor bioavailability, blood levels of Curcumin are very low. Therefore, novel techniques like enzyme inhibitors to slow down metabolization, liposomal formulations, nanoparticles and more stable chemical structural analogues are designed and tested *in vitro* and *in vivo*^8–12^.

The influence of curcumin on multiple cellular pathways like NFkB-, PI3-kinase- or p53-activity leading to inhibition of proliferation and induction of apoptosis was shown in preclinical models of different tumor entities, including pancreatic cancer^13–15^. In addition, impact on formation of reactive oxygen species, regulating RNAs, mitochondrial function, cell membrane and epigenetic changes were described using Curcumin in tumor cell treatment^16–20^. Some *in vitro* studies demonstrated radiosensitization by Curcumin, e.g. in colorectal carcinoma, prostate, lung or head and neck cancer^21–24^, and it is even postulated for pancreatic cancer cells^25^. Besides the effect of Curcumin on radiation efficacy, a sensitization to chemotherapeutic drugs like Gemcitabine was shown *in vitro*^26^.

In this study, the effect of Curcumin on radiosensitivity of the established human pancreatic cancer cell lines Panc-1 and MiaPaCa-2, as well as the effects of a combined treatment on cell cycle distribution, apoptosis and yH2AX-phosphorylation were investigated *in vitro*.

## Results

### Panc-1 cells are more radioresistant than MiaPaCa-2 cells

**Figure 1:**
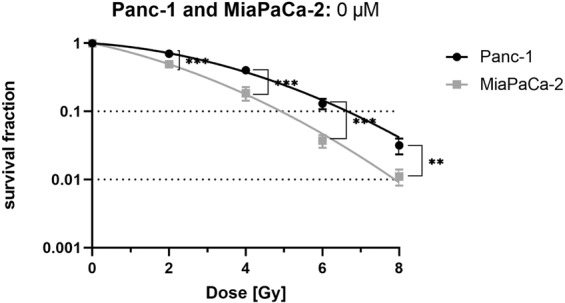
Panc-1 cells are more radioresistant than MiaPaCa-2 cells. Cell survival after irradiation of human pancreatic cancer cells was evaluated by colony forming assay. Points show average, error bars show +/− SD of 4 independent experiments. (*p ≤ 0.05; **p ≤ 0.01; ***p ≤ 0.001).

To investigate the radiosensitivity of pancreatic cancer cell lines Panc-1 and MiaPaCa-2, cells were seeded for CFA and irradiated with doses of 0, 2, 4, 6 and 8 Gy. Panc-1 cells showed compared to MiaPaCa-2 cells a significantly higher survival fraction at every radiation dose (Fig. 1). D_50_ (irradiation dose to reduce the survival rate to 50%) was 3.31 Gy for Panc-1 cells and 1.70 Gy for MiaPaCa-2 cells.

### Curcumin treatment reduces cell survival in both cell lines

**Figure 2:**
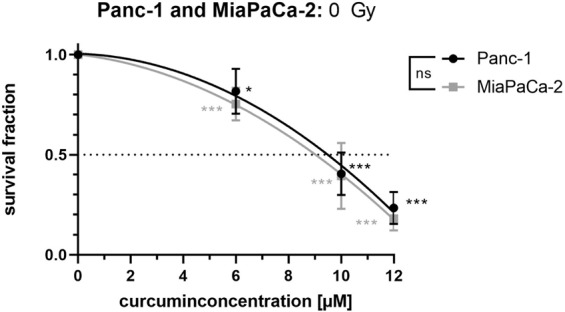
Curcumin reduces survival fraction in a concentration-dependent manner in both cell lines. Human pancreatic cancer cells were seeded for colony forming assay to evaluate cell survival after treatment with different Curcumin concentrations for 24 hours. Points show average, error bars show +/− SD of 4 independent experiments. (*p ≤ 0.05; **p ≤ 0.01; ***p ≤ 0.001).

A single treatment with 6, 10 or 12 µM Curcumin of Panc-1 and MiaPaCa-2 cells without irradiation resulted in a significant concentration-dependent reduction of the survival fraction (Fig. 2). In contrast to their radiosensitivity, the two pancreatic cancer cell lines did not differ significantly in their sensitivity towards Curcumin treatment. A concentration of 9.5 µM and 9.0 µM Curcumin reduced the survival fraction of Panc-1 and MiaPaCa-2 cells to 50% survival, respectively.

### Curcumin sensitizes Panc-1 but not MiaPaca-2 cells to radiation treatment

**Figure 3:**
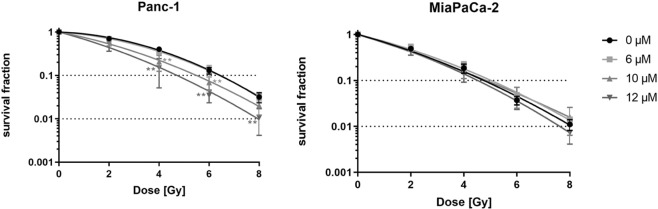
Curcumin sensitizes Panc-1 cells but not MiaPaca-2 cells to irradiation. Radiosensitization was analysed in colony forming assay after treatment with different Curcumin concentrations for 24 hours followed by irradiation with doses of 0, 2, 4 or 8 Gy. Points show average, error bars show +/− SD of 4 independent experiments. (*p ≤ 0.05; **p ≤ 0.01; ***p ≤ 0.001).

The more radioresistant pancreatic cancer cell line Panc-1 was radiosensitized after 24 hours incubation with 10 (4 Gy: p = 0.0048; 6 Gy: p = 0.0096) or 12 µM Curcumin (4 Gy: p = 0.0028; 6 Gy: p = 0.0003; 8 Gy: p = 0.0070) (Fig. 3). An addition of 6 µM Curcumin showed no significant radiosensitization. Sensitization enhancement ratio (SER) for D_50_ was 1.53 for 10 µM and 1.93 for 12 µM Curcumin treatment confirming the radiosensitizing effect of curcumin in Panc-1 cells (Table 1).

**Table 1:** Summary of radiobiological parameters depicted in Fig. 3.

Curcumin [µM]	D_50_ [Gy]	SER(50%)	α [Gy^−1^]	β [Gy^−2^]
**Panc-1**				
0	3.31	1	0.05295	0.0473
6	3.12	1.06	0.0924	0.04171
10	2.16	1.53	0.2566	0.02961
12	1.72	1.93	0.3558	0.02804
**MiaPaCa-2**				
0	1.7	1	0.3636	0.02546
6	1.85	0.92	0.3254	0.02645
10	1.6	1.06	0.4105	0.0135
12	1.64	1.04	0.3714	0.03055

D_50_, dose [Gy] to reduce survival fraction to 50%.

SER, Sensitizing enhancement ratio = D_50_ (irradiation)/D50 (irradiation and curcumin). The SER was calculated in relation to the untreated control cells. A SER greater than 1.20 is indicative of radiosensitization.

α and β values were derived from the linear quadratic equation SF = exp [−α × D − β × D^2^].

In contrast to Panc-1 cells, the MiaPaCa-2 cell line showed no significant radiosensitization in any treatment combination.

### Curcumin increases radiation-induced apoptosis in both cell lines

**Figure 4:**
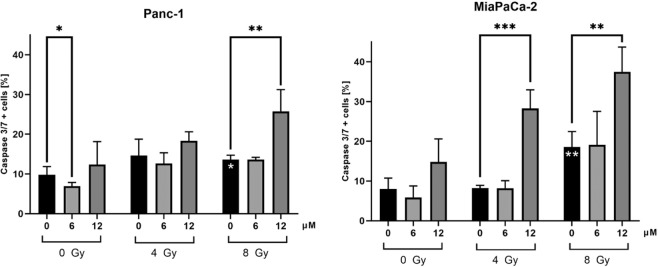
Curcumin increases radiation-induced apoptosis in Panc-1 and MiaPaCa-2 cells. To measure apoptosis induction by irradiation and curcumin, cells were stained with Caspase-3/7 and Sytox 48 hours after irradiation and analysed by flow cytometry. Significance for irradiation alone treatment is illustrated by white stars in the columns. Black stars indicate significance between Curcumin treated cells and untreated cells for each irradiation dose. Columns show average, error bars show +/− SD of 4 independent experiments. (*p ≤ 0.05; **p ≤ 0.01; ***p ≤ 0.001).

To evaluate one possible mechanism of radiosensitization, apoptosis was investigated using Caspase-3/7 staining. As expected, the apoptotic cell fraction increased significantly after irradiation with 8 Gy in both cell lines representing radiation-induced apoptosis (Fig. 4, Panc-1: p = 0.0174; MiaPaCa-2: p = 0.0043). A comparison of the two cell lines revealed a stronger apoptosis induction in MiaPaca-2 cells than in Panc-1 cells after 8 Gy irradiation (Panc-1: 13.6%; MiaPaCa-2: 18.6%; p = 0.049), which might explain the different radiosensitivity observed in CFA.

Curcumin treatment alone did not show apoptosis induction in both cell lines. However, treatment with a low curcumin concentration (6 µM) resulted in a significantly lower fraction of apoptotic cells (p = 0.0449) in the Panc-1 cell line (Fig. 4).

Combined treatment of 8 Gy with 12 µM Curcumin increased the radiation-induced apoptosis significantly in both cell lines (Panc-1 cells: p = 0.0048; MiaPaCa-2 cells: p = 0.0021). In MiaPaCa-2 cells, the enhancement of radiation-induced apoptosis was significant for the combination of 4 Gy and 12 µM as well (p = 0.0001). In both cell lines, a concentration of 6 µM curcumin showed no significant difference compared to the irradiated control without curcumin.

### Curcumin increases radiation-induced DNA damage

**Figure 5:**
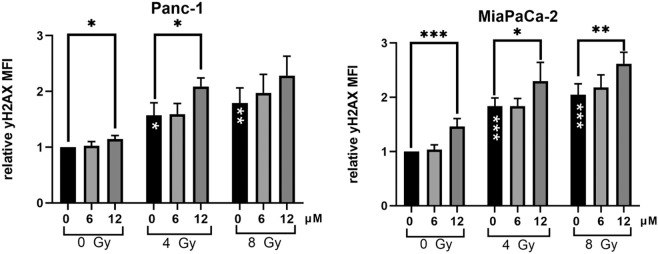
Curcumin treatment enhances radiation-induced DNA-damage. Cells were treated with 0, 6 or 12 µM Curcumin and 24 h later irradiated with 0, 4 or 8 Gy. 30 min after irradiation, yH2AX fluorescence was analysed by flow cytometry. Relative yH2AX-MFI was calculated in relation to untreated cells. Significance for irradiation alone treatment is illustrated by white stars in the bars. Black stars indicate significance between Curcumin treated cells and untreated cells for each irradiation dose. Columns show average, error bars show +/− SD of 3 (Panc-1) or 4 (MiaPaCa-2) independent experiments. (*p ≤ 0.05; **p ≤ 0.01; ***p ≤ 0.001).

Next, we evaluated yH2AX as a marker for DNA-damage, especially DNA double-strand breaks. As expected, irradiation with 4 and 8 Gy increased the yH2AX-MFI in Panc-1 and MiaPaCa-2 cells significantly (Fig. 5). 12 µM Curcumin led to significantly higher relative yH2AX-MFI in unirradiated and irradiated Panc-1 and MiaPaCa-2 cells. Consistent with the other performed experiments in this study, a concentration of 6 µM Curcumin did not significantly change the yH2AX MFI compared to the respective control group.

### Curcumin enhances radiation-induced G2/M arrest

**Figure 6:**
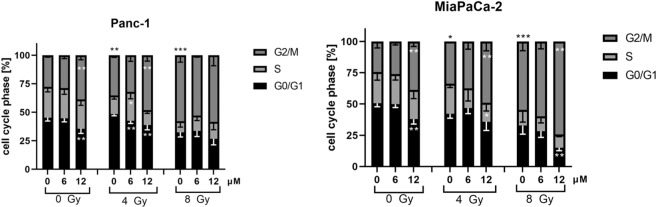
Cell cycle distribution was significantly altered by Curcumin in Panc-1 and MiaPaCa-2 cells. Cells were treated with 0, 6 or 12 µM Curcumin for 24 hours before radiation treatment with 0, 4 or 8 Gy. Cell cycle distribution was evaluated 24 hours after irradiation using propidium iodide staining and analysis by flow cytometry. Significance for sole irradiation treatment is illustrated for G2/M-fraction with black stars above columns. Columns show average, error bars show +/− SD of 4 independent experiments. (*p ≤ 0.05; **p ≤ 0.01; ***p ≤ 0.001).

To determine if Curcumin modifies the effects of irradiation on cell cycle distribution, cell cycle analysis was performed 24 hours after irradiation. As expected, the percentage of Panc-1 and MiaPaCa-2 cells in the G2/M-phase increased significantly after exposure to 4 and 8 Gy irradiation (Fig. 6).

Treatment with 12 µM Curcumin revealed a significantly higher G2/M-fraction of Panc-1 cells in the unirradiated control (p = 0.0021) and also in combination with 4 Gy irradiation (p = 0.0034). Concurrent to the increase of the G2/M-fraction, the G0/G1-fraction was significantly reduced. Irradiation with 4 Gy in combination with 6 µM Curcumin led to a significantly higher portion of cells in the S-phase.

MiaPaCa-2 cells treated with 12 µM Curcumin comprised a significantly higher G2/M-fraction at every combination regime. Accordingly, the G0/G1-phase was reduced significantly by 12 µM Curcumin in unirradiated cells and in cells irradiated with 8 Gy. After a combined treatment with 4 Gy irradiation, the S-phase-fraction was significantly lowered by 12 µM Curcumin.

### Curcumin significantly enhances G2/M-fraction at time of irradiation in Panc-1 cells

**Figure 7:**
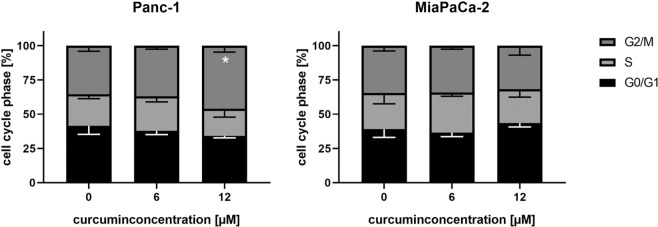
Panc-1 cells show significant G2/M-Arrest 24 h after Curcumin treatment. Pancreatic cancer cells were incubated with different Curcumin concentrations for 24 hours. Cell cycle distribution was measured by flow cytometry using propidium iodide staining. Columns show average, error bars show +/− SD of 4 independent experiments. (*p ≤ 0.05).

As a G2/M cell cycle arrest in non-irradiated pancreatic cancer cells was observed 48 h hours after the addition of 12 µM Curcumin (Fig. 6), we figured out whether the cell cycle distribution was also affected by Curcumin at the time point of irradiation (24 h after Curcumin addition). In line with the 48 h data, 6 µM Curcumin did not show an effect on cell cycle distribution after 24 h in both cell lines (Fig. 7). Most importantly, Panc-1 cells treated with 12 µM Curcumin for 24 hours showed a significantly higher fraction of cells in the radiosensitive G2/M-phase (p = 0.0149). In contrast, cell cycle distribution of MiaPaCa-2 cells was not significantly affected by treatment with 12 µM Curcumin.

This is in line with data from CFA demonstrating radiosensitization in Panc-1 but not in MiaPaCa-2 cells with 12 µM Curcumin. These data suggest that the Curcumin-induced cell cycle arrest in the G2/M-phase, which is the most radiosensitive phase, might be a possible reason for the radiosensitization by Curcumin in Panc-1 cells.

## Discussion

Although there were great advancements in oncology achieved over the last years, prognosis and survival of patients with pancreatic cancer remain very poor. Late diagnosis, early metastasis, bad surgical accessibility and high therapy resistance mark PDAC and limit therapeutic options. Therefore, a variety of agents is evaluated in ongoing *in vitro* and *in vivo* studies to improve the efficacy of RT and to overcome high chemo- and radiation resistance of PDAC. Besides conventional and new chemotherapeutics, promising phytotherapeutics are used in pancreatic cancer research.

One potent example is Curcumin, an orange pigment derived from Curcuma longa root, which is traditionally used in Chinese medicine and showed auspicious results in *in vitro* studies. Besides an observed sensitization to chemotherapy, a radiosensitization of tumor cells is postulated by Curcumin treatment^5,13,27^. In contrast, anti-inflammatory and anti-fibrogenic properties of Curcumin suggest radioprotection of healthy tissues^5^.

In this study, we evaluated radiosensitization effects of Curcumin in two established human pancreatic cancer cell lines. Secondly, we investigated apoptosis induction, yH2AX as an indicator for DNA-double strand breaks and cell cycle distribution to determine the mechanisms underlying radiosensitization.

The efficacy of Curcumin treatment strongly depends on the concentration and also on the formulation used in tumor cell treatment *in vitro*^28^. Most *in vitro* studies in pancreatic cancer cells used concentrations of 5–20 µM to evaluate the impact of stand-alone Curcumin treatment on tumor cell survival and cellular pathways^29–31^. Therefore, in the present study Curcumin concentrations of 6, 10 and 12 µM were chosen to investigate radiosensitizing effects in the pancreatic cancer cell lines Panc-1 and MiaPaCa-2.

Both cell lines showed comparable sensitivity to Curcumin (Fig. 2) with IC_50_ values of 9.5 µM for Panc-1 and 9.0 µM for MiaPaCa-2 cells. Respective other studies, which used a different method to measure cell survival, calculated slightly higher IC_50_ values (e.g. 15 µM^29^ or 25 µM^27^ for Panc-1 cells). In line with the literature^32^. Panc-1 cells revealed higher radioresistance than MiaPaCa-2 cells (Fig. 1).

Most exciting in our study is the difference in radioresponse upon Curcumin treatment between the two pancreatic cancer cell lines Panc-1 and MiaPaCa-2. Whereas the more radioresistant Panc-1 cells showed a significant sensitization to irradiation in CFA, MiaPaCa-2 cells revealed no radiosensitization.

Radiosensitizing effects by Curcumin were observed in various tumor entities. For example, Javvadi *et al*.^33^ observed significant radiosensitization by Curcumin in cervical tumor cell lines. Veeraraghavan *et al*.^25^ first examined the combination of radiation with Curcumin in pancreatic cancer cells. They showed significantly lower cell viability, higher cell death and potentiated apoptosis induction after combination of 100 nM Curcumin with irradiation in Trypan blue exclusion assay, MTT assay and Annexin V-FITC-staining. From their findings they postulated a potential radiosensitization by Curcumin in BxPC-3, Panc-1 and MiaPaCa-2 cells, but they did not perform CFA as the gold standard to determine radiosensitization.

Curcumin has shown multiple effects on cellular pathways that are deregulated in cancer cells, as well as influences on cellular functions like DNA-damage repair, mitrochondrial function or cellular membranes *in vitro*^13^. Especially basal and therapy-induced overactivation of the NFkB-pathway is associated with chemo- and radio-resistance^34^. Down-regulation of NFkB by Curcumin and accompanied apoptosis induction was observed in various tumor models^35^ and discussed as main mechanism of Curcumin’s impact on tumor biology^28,35,36^. *In vitro* experiments with lung cancer cells showed down-regulation of EGFR- and NFkB-AKT-pathway leading to inhibition of proliferation, apoptosis induction and radiosensitization after Curcumin treatment^22,37^. Also in prostate cancer^23^, oesophageal cancer^38^ and in head and neck squamous cell carcinoma cells^24^ radiosensitization by Curcumin was observed and associated with its impact on NFkB- and EGFR-pathways. In pancreatic cancer cell lines radiation-induced NFkB activity was inhibited by Curcumin consequential leading to a significantly higher apoptosis induction^25^. Therefore, Veeraghavan *et al*. postulated a down-regulation of the NFkB-pathway as a cause for radiosensitization in pancreatic cancer cells.

An association between cyclooxygenase-2 (COX-2) expression and apoptosis induction by Curcumin was found for lung and pancreatic cancer cell lines, revealing increased Curcumin sensitivity of high COX-2 expressing cells compared to low-expressing ones^27,28^. In line with these data, we observed no significant apoptosis induction by a single treatment with Curcumin in the COX-2 low expressing cell lines Panc-1 and MiaPaCa-2 cells.

Interestingly, in combination with irradiation, Curcumin enhanced radiation-induced apoptosis in both cell lines. Since we observed radiosensitization in Panc-1 cells, but not in MiaPaCa-2 cells without significant difference in apoptosis induction by Curcumin treatment between the two cell lines, we concluded, that other mechanisms for radiosensitization have to be evaluated.

Since the therapeutic effects of RT are based predominantly on the induction of DNA-double strand breaks^39^, we analyzed yH2AX-phosphorylation as an indicator of DNA-damage. Some studies showed that Curcumin inhibits DNA-damage repair. For example Sahu *et al*. found an increase of phosphorylated yH2AX in BxPC-3 cells after treatment with 2.5 µM Curcumin^40^. The combination of Curcumin with a DNA-damage-inducing treatment like chemotherapy or irradiation was evaluated in human myeloic cancer cells showing significant enhancement of DNA double-strand breaks by the combination of etoposide and Curcumin^41^. In line with the literature, we found increased yH2AX phosphorylation after single treatment with a high Curcumin concentration in Panc-1 and MiaPaca-2 cells. The combination of Curcumin with irradiation further increased yH2AX phosphorylation in both cell lines.

The G2/M phase is known to be the most sensitive to irradiation treatment^42^. Furthermore, DNA damage or blocked DNA replication forks lead to a G2/M arrest^43^. A single treatment with Curcumin has been shown to induce cell cycle arrest in G2/M-phase *in vitro*^40^. As an example, Subramaniam *et al*.^19^ found a G2/M arrest for Panc-1 and MiaPaCa-2 cells after incubation with 30 µM Curcumin for 24 hours. Based on these data, we analyzed cell cycle distribution of curcumin-treated human pancreatic cancer cells as a potential mechanism of radiosensitization.

Comparable to Veeraraghavan *et al*.^25^, a pre-treatment of Panc-1 and MiaPaca-2 cells with Curcumin led to a higher amount of cells in G2/M-phase 24 hours after irradiation. But more interestingly, we found a significant increase of the G2/M-fraction at the irradiation time point only in Panc-1 but not in MiaPaCa-2 cells after treatment with Curcumin. The Curcumin-induced G2/M phase arrest in the more radioresistant Panc-1 cells might be the reason for the observed radiosensitizing effects in CFA for Panc-1 cells.

On the one hand, Curcumin sensitizes cancer cells to anti-tumor treatment *in vitro;* on the other hand, anti-inflammatory properties postulate lower therapy side effects under concomitant phytotherapeutical treatment. Oral intake of Curcumin showed for example, significantly reduced bowel toxicity after abdominal irradiation in rats and lower radiation-induced pneumonitis after irradiation of rat lungs^44^. Wound-healing was significantly accelerated in Curcumin pre-treated mice undergoing fractionated RT after surgery^45^. In humans, oral doses up to 12 g daily showed no toxic side effects and were well tolerated^46^. A randomized treatment of breast cancer patients medicated with 6 g Curcumin daily parallel to radiation therapy showed significant reduction of radiation dermatitis severity and moist desquamation, but no significant effects on pain, redness or attendant symptoms like nausea or fatigue^47^. CT-evaluated body consumption and weight loss were evaluated in patients with advanced pancreatic cancer receiving 8 g Curcumin per day. No significant difference compared to the control group was found^48^. Considering the metabolic rate of curcumin in human, an oral intake 6 to 8 hours before radiotherapy would be suggested as unformulated curcumin reached the maximum blood concentration at that time^49^.

However, caused by chemistry and pharmacology, Curcumin has a very low bioavailability, chemical instability and fast metabolism. Blood levels after oral intake of 8 g Curcumin daily remained very low and did not outrange a concentration of 40 ng/ml equivalent to only 0.11 µM^6^. Actually, other studies detected no Curcumin in the blood of humans after a single oral intake^50^. Compared to the effective tumor-suppressive and radiosensitizing concentrations used *in vitro*, blood levels in humans are more than 100 times lower.

A major characteristic of PDAC is the presence of a dense desmoplastic tumor stroma with a volume up to 70% of the total tumor volume, which contains cellular, acellular and biophysical components. The dysplastic stroma serves as a physical and biological barrier for drug delivery, an unfavorable pharmacokinetic and pharmacodynamic profile resulting in a high drug resistance. Therefore, mechanisms such as vasculature permeability, blood vessel patency, drug activation/degradation enzymes or target specific biological factors are develped to improve intratumoral distribution and bioavailability aiming to overcome drug resistance^51^. Besides the stromal barrier, a highly heterogeneous and genetically diversity as well as the tumor microenvironment contribute to high therapeutic resistance in PDAC. Subtype-driven therapy decisions are not yet implemented in clinical routine and are restricted to very few molecular alterations. Although targeting of various stromal components and pathways was considered a promising therapeutic strategy, none of the efforts had yet led to efficacious and approved therapies in patients with PDAC^52^.

To address the poor bioavailability and increase blood concentrations of Curcumin for an more effective treatment with Curcumin in cancer patients, several approaches and chemical modifications like inhibition of metabolization, better absorption through covering in nanoparticles^12^ or liposomes^9^, structural analoges^17^, phospholipid complexes or combinations with other dietary components are investigated *in vitro* and *in vivo*^53^. Small studies with healthy volunteers show higher blood levels of curcumin and its metabolites after oral intake of micelles or phospholipid complex formulations of curcumin. Besides the oral intake of curcumin, liposomal formulations are developed and evaluated for parenteral use. In cancer therapy especially nanoparticles are used^54^. Experiments with nanoparticles or carrier complexes in pancreatic cancer models are still ongoing^55^, while erythrocyte membrane cloaked curcumin-loaded nanoparticles showed tumor cell apoptosis as well as reduction in tumor size and tumor mass of hepatocellular tumors in mice without any observable toxicity^56^. Considering future research directions an individualization of stromal targeting and translational clinical trials comparing preclinical approaches in patient-derived xenografts with patients’ response to personalize therapies are recommended^52^.

## Conclusion

Treatment with Curcumin showed significant effects on survival, apoptosis, DNA-double-strand breaks and cell cycle distribution in pancreatic cancer cells *in vitro*. Only in the more radioresistant cell line Panc-1 a dose-dependent radiosensitization was achieved by Curcumin treatment. A Curcumin-induced cell cycle arrest in the radiosensitive G2/M phase is assumed as a possible reason for radiosensitization. In conclusion, our results warrant further studies to understand the detailed molecular mechanisms leading to radiosensitization by Curcumin and in the long term to enable an optimized and individualized treatment concept for pancreatic cancer patients.

## Material and Methods

### Reagents and treatment

Curcumin powder (Sigma-Aldrich, St. Louis, USA) was solved freshly in 100% DMSO to get a 50 mM stock solution and was then diluted with warm cell culture medium to concentrations of 12, 10 or 6 µM Curcumin. The highest end-concentration of DMSO was 0.02% (≙12 µM Curcumin). Cells were incubated with Curcumin 24 hours before irradiation and if not described otherwise, cell culture medium was changed by drug-free medium directly after radiation treatment.

### Cells and cell culture

Panc-1 and MiaPaCa-2 (DSMZ, Braunschweig, Germany) cells were cultured in Dulbecco’s Modified Eagle’s high glucose culture medium with 10% FCS and 1% Penicillin-Streptomycin solution. Cells were routinely checked for mycoplasma contamination by MycoAlert Mycoplasma Detection Kit (Lonza Group, Basel, Switzerland).

### Colony forming assay

Colony forming assay (CFA) was performed to measure radiosensitivity and sensitivity towards Curcumin. Cells were seeded in 12-well plates and 24 hours later treated with different Curcumin concentrations or drug-free cell culture medium (control group). Again 24 hours later cells were irradiated at RS225A irradiation device (Gulmay Medical Ltd/Xstrahl, Camberley, UK) with doses of 0, 2, 4, 6 or 8 Gy. After irradiation and medium change, cells grew 11 (MiaPaCa-2) or 12 (Panc-1) days and were then fixed with ice-cold methanol and stained with 0.1% crystal violet. Colonies were counted with the colony counter GelCount (Oxford Optronics). Survival curves of irradiated cells were fitted to the linear-quadratic model using GraphPad Prism (San Diego, USA). Survival curves of Curcumin-treated, non-irradiated cells were plotted by second order polynomial function.

### Apoptosis

Cells were treated with 0, 6 or 12 µM Curcumin for 24 hours and then irradiated with 0, 4 or 8 Gy at RS225A irradiation device. Apoptotic cells were stained 48 hours after irradiation using CellEvent Caspase 3/7 assay in combination with SYTOX AADvanced dead cell stain (both ThermoFisher Scientific). FACS-analysis was performed using FACSCalibur flow cytometer (BD Biosciences, San Jose, CA, USA). Not-stained, but Curcumin-treated cells were used to exclude false-positive signals caused by autofluorescence of Curcumin.

### Cell cycle

To analyze cell cycle distribution, cells were treated with Curcumin and 24 hours later irradiated with 0, 4 or 8 Gy. Cells were fixed with 70% cold ethanol and stained with propidium iodide (Invitrogen, ThermoFisher Scientific, USA) in the presence of RNAse at two different time points, directly at the time point of irradiation or 24 hours after irradiation. Results from flow cytometry (FACSCalibur flow cytometer, BD Biosciences, San Jose, CA, USA) were evaluated with ModFit-Software (Verity software house Inc).

### yH2AX

As a marker for DNA-double-strand-breaks, yH2AX-fluorescence intensity was measured using flow cytometry (FACSCalibur flow cytometer BD Biosciences, San Jose, CA, USA). Therefore, cell populations were collected and subsequent fixed with 1% PFA and −20 °C cold 70% ethanol 30 minutes after irradiation treatment with 0, 4 or 8 Gy. For analysis, cells were stained with primary (anti-phospho-histone H2A.X Ser139 monoclonal mouse antibody, Invitrogen, ThermoFisher Scientific, USA) and secondary antibody (Alexa 488 goat anti-mouse F(ab)2, Invitrogen, ThermoFisher Scientific, USA).

Results were analyzed with BD CellQuest Pro (BD Biosciences, San Jose, USA) and relative mean fluorescence intensity (MFI) values were calculated for each independent experiment. As described for apoptosis, curcumin autofluorescence was excluded before performing yH2AX-analysis.

### Statistical analysis

All experiments were repeated at least three or four times. To evaluate statistical significance, GraphPad Prism Software (San Diego, USA) was used and results were calculated as mean ± SD. If not described otherwise, analysis was done with unpaired t-test comparing the treated group with untreated control.

## Data Availability

All data supporting findings of this study are available within the article or from the corresponding author upon request.
